# Synthesis and crystal structure of 2-(1,3-dithietan-2-yl­idene)cyclo­hexane-1,3-dione

**DOI:** 10.1107/S2056989022009872

**Published:** 2022-10-13

**Authors:** Sabah Kellou, Ouarda Brihi, Souheyla Chetioui, Lemallem Salah Eddine, Fiala Abdelali, Ali Boudjada

**Affiliations:** a Laboratoire de Cristallographie, Département de Physique, Université des Frères Mentouri de Constantine-1, 25000 Constantine, Algeria; b Unité de Recherche de Chimie de l’Environnement et Moléculaire Structurale (URCHEMS), Département de Chimie, Université des Frères Mentouri de Constantine-1, 25000 Constantine, Algeria; c Faculté de Technologie, Université Mohamed Boudiaf, M’sila, Algeria; University of Aberdeen, Scotland

**Keywords:** ketene di­thio­acetal, hydrogen bond, Hirshfeld surface analysis, crystal structure

## Abstract

The title compound, C_8_H_8_O_2_S_2_, contains a cyclo­hexane-1,3-dione ring, which has a twist-boat conformation. The C_2_S_2_ ring is close to planar (r.m.s. deviation = 0.023 Å) and the dihedral angle between the mean planes of the cyclo­hexane and 1,3-dithietane rings is 9.1 (3)°. Short intra­molecular S⋯O contacts occur [2.719 (5) and 2.740 (5) Å]. In the crystal, the mol­ecules are linked by weak C—H⋯S hydrogen bonds and short [3.165 (5) Å] S⋯O contacts, forming (010) layers. The prevalence of these inter­actions is illustrated by an analysis of the three-dimensional Hirshfeld surface and by two-dimensional fingerprint plots.

## Abstract 2

In the title compound, the dihedral angle between the mean planes of the cyclo­hexane and 1,3-dithietane rings is 9.1 (3)°. A short S⋯O contact is observed in the crystal.

## 1. Chemical context

Ketene di­thio­acetals are useful inter­mediates in organic synthesis and have been used for the preparation of heterocyclic compounds (Kolb, 1990 ▸; Ila *et al.*, 2001 ▸). The synthesis of tri­fluoro­methyl ketene di­thio­acetals has applications in the field of pharmaceuticals and agrochemicals (Gouault-Bironneau *et al.*, 2012 ▸; Timoshenko & Portella, 2009 ▸). The functionalization of ketene di­thio­acetals provides more powerful tools for the development of new inter­mediates (Wang *et al.*, 2011 ▸; Gao *et al.*, 2010 ▸; Hu *et al.*, 2012 ▸). The direct formation of a C—C bond has been carried out by reacting a cyano ketene di­thio­acetal and Morita–Baylis–Hillman (MBH) alcohols resulting from the reaction of acrylo­nitrile and aryl aldehydes. This reaction led to the corresponding 1,4-penta­diene deriv­atives (Zhao *et al.*, 2007 ▸). Fiala *et al.* (2007 ▸) have studied the inhibitive action of some synthetic ketene di­thio­acetal deriv­atives towards the corrosion of copper in aerated nitric acid solutions. They concluded that these compounds are good inhibitors of copper corrosion in this medium. In the present study, we report the synthesis, crystal structure and Hirshfeld surface analysis of the new title 1,3-di­thian-2-yl­idene derivative, C_8_H_8_O_2_S_2_, (I). 
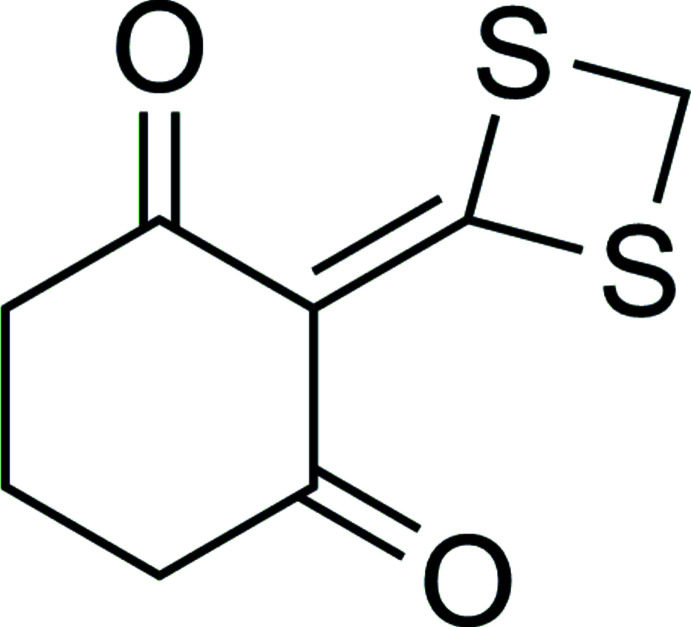


## 2. Structural commentary

**Figure 1:**
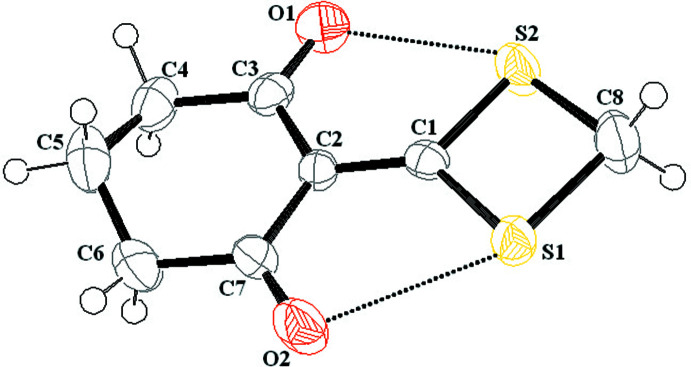
The mol­ecular structure of (I) with displacement ellipsoids drawn at the 50% probability level. The short intra­molecular S⋯O contacts are shown as dashed lines.

In the mol­ecular structure of (I), the cyclo­hexane and dithietane rings are linked by a C=C bond of 1.364 (8) Å (Fig. 1 ▸). The cyclo­hexane-1,3-dione ring adopts a twist-boat conformation, as seen in related compounds (Kuppan Chandralekha *et al.*, 2016 ▸; Liu *et al.*, 2011 ▸). Atom C5 is displaced by 0.627 (8) Å with respect to the C2/C3/C4/C6/C7 mean plane, similar to the value observed for 2-[chloro­(4-meth­oxy­phen­yl)meth­yl]-2-(4-meth­oxy­phen­yl)-5,5-di­methyl­cyclo­hexane-1,3-dione (Saloua Chelli *et al.*, 2016 ▸). The largest endocyclic angle in the cyclo­hexane ring [C7—C2—C3 = 123.2 (6)°] is located opposite the dithiethan ring and the largest exo-cyclic angle (C6—C7—O2) is 122.3 (5)°. A difference of 1.3° is observed between the angles located on either side of the C1=C2 double bond. In the C_2_S_2_ ring, the C1—S1 and C1—S2 bond lengths are indistinguishable at 1.716 (6) Å whereas the C8—S1 and C8—S2 bond lengths differ slightly [1.819 (7) and 1.801 (7) Å, respectively]. The mol­ecule has local *C_s_* symmetry with a non-crystallographic mirror plane passing through atoms C8, C1, C2 and C5. The dihedral angle between the cyclo­hexane (all atoms) and dithietane rings is 9.1 (3)° and short intra­molecular S1⋯O2 [2.719 (5) Å] and S2⋯O1 [2.740 (5) Å] contacts are observed (Fig. 1 ▸).

## 3. Supra­molecular features

**Figure 2:**
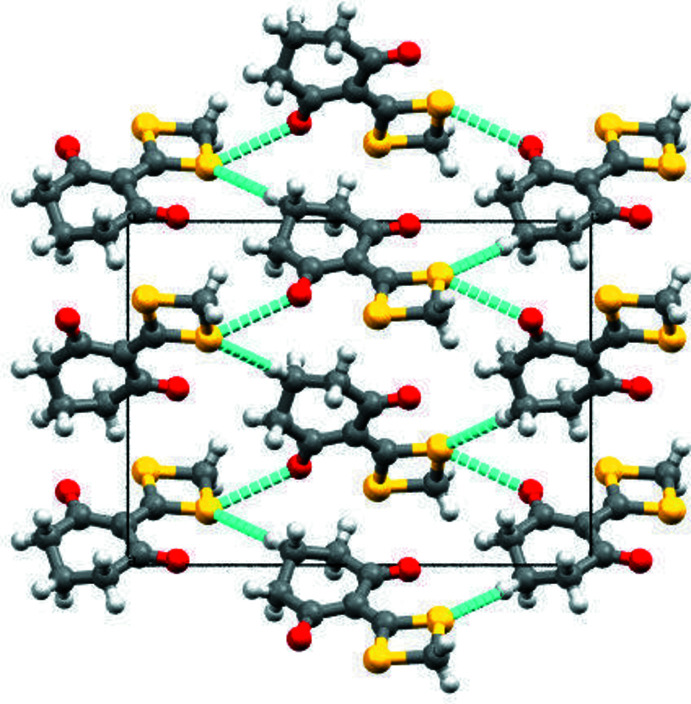
Structure of (I) viewed along the [010] direction, showing the infinite layers propagating parallel to the *ac* plane. The C—H⋯S and short S⋯O contacts are shown as blue dashed lines.

In the crystal, the mol­ecules stack head-to-tail along the *b*-axis direction. The mol­ecules are linked by C5—H5*A*⋯S2 hydrogen bonds (Table 1 ▸) and short [3.165 (5) Å compared to a van der Waals separation of 3.32 Å] S2⋯O2^ii^ [symmetry code: (ii) 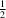 − *x*, *y*, 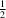 + *z*] contacts, forming (010) layers (Fig. 2 ▸).

## 4. Hirshfeld surface analysis

**Figure 3:**
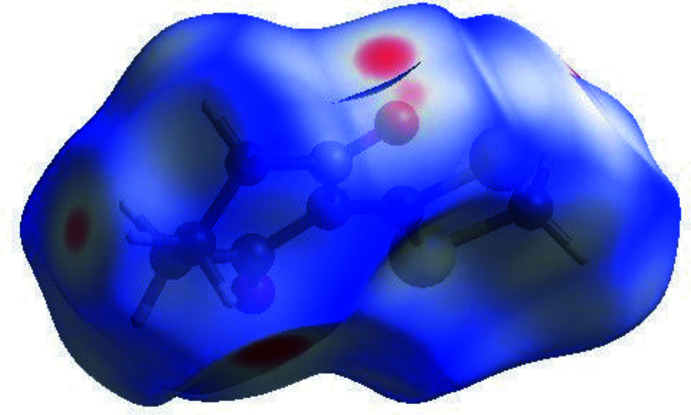
Hirshfeld surface for (I) scaled from −0.16 (red) a.u. to 1.09 (blue) a.u.

**Figure 4:**
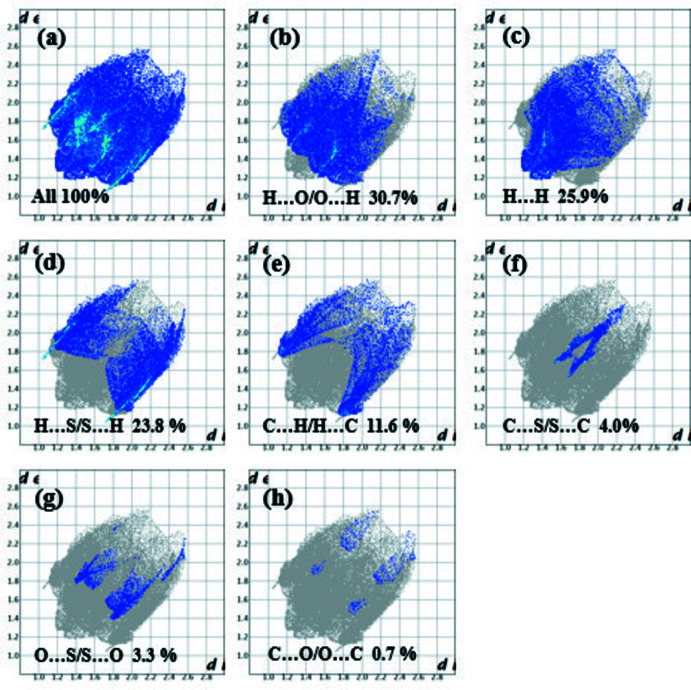
Two-dimensional finger print plots for (I): (*a*) overall, and delineated into contributions from different contacts: (*b*) H⋯O/O⋯H, (*c*) H⋯H, (*d*) H⋯S/S⋯H, (*e*) C⋯H/H⋯C, (*f*) C⋯S/S⋯C, (*g*) O⋯S/S⋯O and (*h*) C⋯O/O⋯C.

The nature of the inter­molecular inter­actions in (I) has been computed by *CrystalExplorer17.5* (Turner *et al.*, 2017 ▸), using Hirshfeld surface (HS) analysis (Spackman & Jayatilaka, 2009 ▸) and two-dimensional fingerprint plots (McKinnon *et al.*, 2007 ▸). The *d*_norm_ plot (Fig. 3 ▸) shows red spots corresponding to the C5—H5*A*⋯S2 hydrogen bond and short S2⋯O2 contact. A list of the relative percentage contributions of the close contacts to the HS of (I) are given in Table 2 ▸ and the overall two-dimensional fingerprint plot is shown in Fig. 4 ▸ *a*. A contribution of 30.7% was found for the H⋯O/O⋯H inter­actions, representing the largest contribution; these contacts are represented by the spikes in the top left (*d*_e_ > *d*_i_, H⋯O, 14.3%) and bottom right (*d*_e_ < *d*_i_, O⋯H, 16.5%) of Fig. 4 ▸ *b*. Inter­actions of the type H⋯H appear in the middle of the scattered points in the fingerprint plots with a pair of spikes at *d*_e_ + *d*_i_ = 2.5 Å and comprise 25.9% of the entire surface (Fig. 4 ▸ *c*); the van der Waals radius for this inter­action is 2.4 Å, which means it is a weak inter­action. The S⋯H/H⋯S contacts (Fig. 4 ▸ *d*), which account for 23.8% of the Hirshfeld surface, are displayed on the fingerprint plot as a pair of long spikes at *d*_e_ + *d*_i_ = 2.7Å. This distance differs by 0.3 Å from the sum of the van der Waals radii, which means it is the strongest inter­action present. The S⋯C/C⋯S (4.0%, Fig. 4 ▸ *f*) and S⋯O/O ⋯S (3.3%, Fig. 4 ▸ *g*) contacts are seen as pairs of spikes at *d*_e_ + *d*_i_ = 3.2 and 3.05 Å, respectively. These distances are shorter than the sums of the van der Waals radii of 3.5 and 3.32 Å, respectively. The C⋯O/O⋯C inter­actions make a contribution of 0.7% to the Hirshfeld surface (Fig. 4 ▸ *h*), their inter­atomic distances (*d*_e_ + *d*_i_ = 3.3 Å) being larger than the sum of the van der Waals radius (3.22 Å), so this inter­action is very weak in this structure. The fingerprint plot corresponding to C⋯H/H⋯C contacts (Fig. 4 ▸ *e*) shows a fin-like distribution of points with the edges at *d*_e_ + *d*_i_ = 2.8 Å.

## 5. Database survey

A search of the Cambridge Structural Database (CSD, Version 5.43, last update March 2022 ; Groom *et al.*, 2016 ▸) for the 1,3-dithietane fragment yielded three relevant hits. These are di­spiro­[1,3-dithietane-2,2′:4,2′′-diadamantane] (CSD refcode AFECAP; Linden *et al.*, 2002 ▸), *trans*-2,4-bis­(isoprop­yl)-2,4-bis­[(2-methyl-1-thioxo)propyl­sulfan­yl]-1,3-dithietane (HUZ­HOZ; Mahjoub *et al.*, 2003 ▸) and 2-(nitro­methyl­ene)-1,3-dithietane (WOCQEK; Shanmuga Sundara Raj *et al.*, 2000 ▸): in these compounds the dithietane ring is planar. In (I), the angles C1—S1—C8 and S1—C8—S2 are 82.7 (3) and 93.6 (3)°, respectively, similar to the values observed for the aforementioned compounds, *viz*. 85.76 and 94.24°, 85.40 and 94.60°, 82.8 and 94.00° for AFECAP, HUZHOZ and WOCQEK, respectively. A search for the cyclo­hexane-1,3-dione fragment revealed over 30 hits. The most relevant structures are 2-(phenyl­amino­methyl­idene)cyclo­hexane-1,3-dione (ISUQAO; Kettmann *et al.*, 2004 ▸), (*E*)-5,5-dimethyl-2- [3-(4- nitro­phen­yl)allyl­idene]cyclo­hexane-1,3-dione (VUGVUQ; Jae Kyun Lee *et al.*, 2015 ▸), 2-[chloro­(4-meth­oxy­phen­yl)meth­yl]-2-(4-meth­oxy­phen­yl)-5,5-di­methyl­cyclo­hexane-1,3-dione (TAC­ZIJ; Saloua Chelli *et al.*, 2016 ▸) and 2-{(1S*,2S*)-2-[(*E*)-(2,4-di­hydroxy­benzyl­idene)amino]­cyclo­hex­yl}isoindoline-1,3-di­one (EVABIN; Liu *et al.*, 2011 ▸). The cyclo­hexane ring adopts a chair conformation in all five of these compounds, as in the title compound.

## 6. Synthesis and crystallization

Potassium carbonate (0.3 mol, 42 g) in DMF (50 ml) was well stirred at room temperature. To this mixture, cyclo­hexane-1,3-dione (0.1 mol) was added and the resultant solution stirred at room temperature for 20 min. Carbon di­sulfide (0.15 mol, 9.0 ml) was then added in one lot. The reaction mixture was stirred and kept for 10 min at room temperature. Di­iodo­methane (0.12 mol) was added dropwise over 20 min and the reaction mixture stirred for 7 h at room temperature. Ice–water (500 ml) was added to the reaction mass, the solid was filtered and washed with water, dried and recrystallized from ethanol solution to give (I) in the form of colourless plates. Yield 81%; m.p. 487 K; UV (H_2_O) λ_max_, 335 nm (ɛ 18760); IR (KBr, cm^−1^): 1640 (C=O), ^1^H NMR (CDCl_3_) δ (ppm): 4.35 (*s*, 2H, CH_2_—S), 2.52 (*t*, *J* = 6.5 Hz, 4H, CH_2_—CH_2_—CH_2_), 1.97 (*q*, *J* = 6.5 Hz, 2H, CH_2_—CH_2_—CH_2_); ^13^C NMR (CDCl_3_) δ (ppm): 197.28 (CO),189.73 (C=C—S), 119.93 (C=C—S), 37.31 (CH_2_—CH_2_—CH_2_), 33.39 (CH_2_—S), 18.62 (CH_2_—CH_2_—CH_2_).

## 7. Refinement

Crystal data, data collection and structure refinement details for the title compound are summarized in Table 3 ▸. H atoms were positioned geometrically with C—H = 0.97 Å and refined as riding with *U*_iso_(H) = 1.2*U*_eq_(C).

## Supplementary Material

Crystal structure: contains datablock(s) I. DOI: 10.1107/S2056989022009872/hb8028sup1.cif

Structure factors: contains datablock(s) I. DOI: 10.1107/S2056989022009872/hb8028Isup2.hkl

Supporting information file. DOI: 10.1107/S2056989022009872/hb8028Isup3.cml

CCDC reference: 2211891

Additional supporting information:  crystallographic information; 3D view; checkCIF report

## Figures and Tables

**Table 1:** Hydrogen-bond geometry (Å, °)

*D*—H⋯*A*	*D*—H	H⋯*A*	*D*⋯*A*	*D*—H⋯*A*
C5—H5*A*⋯S2^i^	0.97	2.87	3.844 (8)	178

Symmetry code: (i) 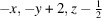 .

**Table 2:** Relative percentage contributions of the close contacts to the Hirshfeld surface of the title compound

Contact type	Percentage contribution
O⋯H/H⋯O	30.7
H⋯H	25.9
S⋯H/H⋯S	23.8
C⋯H/H⋯C	11.6
S⋯C/S⋯C	4.0
S⋯O/O⋯S	3.3
C⋯O/O⋯C	0.7

**Table 3:** Experimental details

Crystal data	
Chemical formula	C_8_H_8_O_2_S_2_
*M*_r_	200.28
Crystal system, space group	Orthorhombic, *P* *c* *a*2_1_
Temperature (K)	296
*a*, *b*, *c* (Å)	10.7521 (14), 5.5245 (9), 14.480 (2)
*V* (Å^3^)	860.1 (2)
*Z*	4
Radiation type	Mo *K*α
μ (mm^−1^)	0.57
Crystal size (mm)	0.13 × 0.06 × 0.01

Data collection	
Diffractometer	Bruker APEXII
Absorption correction	Multi-scan (*SADABS*; Krause *et al.*, 2015 ▸)
*T*_min_, *T*_max_	0.960, 0.994
No. of measured, independent and observed [*I* > 2σ(*I*)] reflections	3646, 1881, 1230
*R*_int_	0.044
(sin θ/λ)_max_ (Å^−1^)	0.649

Refinement	
*R*[*F* ^2^ > 2σ(*F* ^2^)], *wR*(*F* ^2^), *S*	0.051, 0.094, 1.00
No. of reflections	1881
No. of parameters	109
No. of restraints	1
H-atom treatment	H-atom parameters constrained
Δρ_max_, Δρ_min_ (e Å^−3^)	0.34, −0.30
Absolute structure	Flack *x* determined using 396 quotients [(*I* ^+^)−(*I* ^−^)]/[(*I* ^+^)+(*I* ^−^)] (Parsons *et al.*, 2013 ▸)
Absolute structure parameter	0.04 (8)

Computer programs: *APEX2* and (Bruker, 2014 ▸), *SHELXT* (Sheldrick, 2015*b* ▸), *SHELXL2014/7* (Sheldrick, 2015*a* ▸) and *ORTEP-3 for Windows* and *WinGX* publication routines (Farrugia, 2012 ▸).

## supplementary crystallographic information

### Crystal data

**Table:**

C_8_H_8_O_2_S_2_	*F*(000) = 416
*M**_r_* = 200.28	*D*_x_ = 1.547 Mg m^−^^3^
Orthorhombic, *P**c**a*2_1_	Mo *K*α radiation, λ = 0.71073 Å
Hall symbol: P 2c -2ac	Cell parameters from 772 reflections
*a* = 10.7521 (14) Å	θ = 3.7–23.4°
*b* = 5.5245 (9) Å	µ = 0.57 mm^−^^1^
*c* = 14.480 (2) Å	*T* = 296 K
*V* = 860.1 (2) Å^3^	Plate, colorless
*Z* = 4	0.13 × 0.06 × 0.01 mm

### Data collection

**Table:**

Bruker APEXII diffractometer	1881 independent reflections
Radiation source: fine-focus sealed tube	1230 reflections with *I* > 2σ(*I*)
Graphite monochromator	*R*_int_ = 0.044
CCD rotation images, thick slices scans	θ_max_ = 27.5°, θ_min_ = 2.8°
Absorption correction: multi-scan (SADABS; Krause *et al.*, 2015)	*h* = −9→13
*T*_min_ = 0.960, *T*_max_ = 0.994	*k* = −4→7
3646 measured reflections	*l* = −18→18

### Refinement

**Table:**

Refinement on *F*^2^	Secondary atom site location: difference Fourier map
Least-squares matrix: full	Hydrogen site location: inferred from neighbouring sites
*R*[*F*^2^ > 2σ(*F*^2^)] = 0.051	H-atom parameters constrained
*wR*(*F*^2^) = 0.094	*w* = 1/[σ^2^(*F*_o_^2^) + (0.0343*P*)^2^] where *P* = (*F*_o_^2^ + 2*F*_c_^2^)/3
*S* = 1.00	(Δ/σ)_max_ < 0.001
1881 reflections	Δρ_max_ = 0.34 e Å^−^^3^
109 parameters	Δρ_min_ = −0.29 e Å^−^^3^
1 restraint	Absolute structure: Flack *x* determined using 396 quotients [(*I*^+^)-(*I*^-^)]/[(*I*^+^)+(*I*^-^)] (Parsons *et al.*, 2013)
0 constraints	Absolute structure parameter: 0.04 (8)
Primary atom site location: structure-invariant direct methods	

### Special details

**Table:**

Geometry. All esds (except the esd in the dihedral angle between two l.s. planes) are estimated using the full covariance matrix. The cell esds are taken into account individually in the estimation of esds in distances, angles and torsion angles; correlations between esds in cell parameters are only used when they are defined by crystal symmetry. An approximate (isotropic) treatment of cell esds is used for estimating esds involving l.s. planes.

### Fractional atomic coordinates and isotropic or equivalent isotropic displacement parameters (Å^2^)

**Table:**

	*x*	*y*	*z*	*U*_iso_*/*U*_eq_	
S1	0.27332 (13)	0.3620 (3)	0.53923 (11)	0.0371 (5)	
S2	0.16018 (17)	0.5982 (3)	0.67352 (10)	0.0430 (5)	
O1	0.0128 (4)	0.9617 (7)	0.6058 (4)	0.0457 (16)	
O2	0.2139 (4)	0.5407 (8)	0.3699 (3)	0.0520 (19)	
C1	0.1711 (5)	0.5968 (10)	0.5553 (4)	0.0273 (19)	
C2	0.1164 (5)	0.7427 (12)	0.4911 (4)	0.0260 (17)	
C3	0.0319 (5)	0.9299 (10)	0.5239 (5)	0.034 (2)	
C4	−0.0307 (6)	1.0849 (12)	0.4519 (5)	0.045 (2)	
C5	−0.0404 (7)	0.9587 (14)	0.3579 (6)	0.060 (3)	
C6	0.0814 (7)	0.8675 (13)	0.3269 (4)	0.058 (3)	
C7	0.1416 (6)	0.7004 (12)	0.3941 (4)	0.034 (2)	
C8	0.2644 (7)	0.3440 (12)	0.6645 (5)	0.050 (3)	
H4A	−0.11352	1.12679	0.47307	0.0542*	
H4B	0.01593	1.23405	0.44470	0.0542*	
H5A	−0.07254	1.07203	0.31262	0.0722*	
H5B	−0.09848	0.82483	0.36241	0.0722*	
H6A	0.07063	0.78338	0.26865	0.0697*	
H6B	0.13618	1.00398	0.31609	0.0697*	
H8A	0.34374	0.37194	0.69448	0.0598*	
H8B	0.22773	0.19389	0.68617	0.0598*	

### Atomic displacement parameters (Å^2^)

**Table:**

	*U*^11^	*U*^22^	*U*^33^	*U*^12^	*U*^13^	*U*^23^
S1	0.0410 (8)	0.0394 (9)	0.0308 (8)	0.0115 (8)	0.0003 (9)	0.0007 (9)
S2	0.0561 (11)	0.0509 (10)	0.0219 (7)	0.0117 (9)	0.0005 (8)	−0.0001 (10)
O1	0.052 (3)	0.045 (3)	0.040 (2)	0.011 (2)	0.006 (3)	−0.010 (3)
O2	0.070 (4)	0.058 (3)	0.028 (3)	0.020 (3)	0.008 (3)	−0.001 (2)
C1	0.026 (3)	0.028 (4)	0.028 (3)	−0.006 (2)	0.002 (3)	0.000 (3)
C2	0.027 (3)	0.026 (3)	0.025 (3)	0.002 (3)	−0.001 (3)	0.000 (3)
C3	0.026 (3)	0.032 (4)	0.044 (4)	−0.003 (3)	0.001 (3)	0.004 (3)
C4	0.039 (4)	0.038 (4)	0.059 (4)	0.003 (3)	−0.007 (4)	0.009 (4)
C5	0.064 (6)	0.069 (6)	0.048 (4)	0.013 (4)	−0.014 (4)	0.016 (5)
C6	0.061 (5)	0.072 (5)	0.041 (4)	0.020 (4)	0.010 (4)	0.024 (4)
C7	0.035 (4)	0.039 (4)	0.028 (3)	−0.002 (3)	0.003 (3)	0.005 (3)
C8	0.064 (5)	0.052 (5)	0.034 (4)	0.014 (3)	−0.006 (4)	0.006 (4)

### Geometric parameters (Å, º)

**Table:**

S1—C1	1.716 (6)	C5—C6	1.473 (11)
S1—C8	1.819 (7)	C6—C7	1.489 (9)
S2—C1	1.716 (6)	C4—H4A	0.9700
S2—C8	1.801 (7)	C4—H4B	0.9700
O1—C3	1.216 (9)	C5—H5A	0.9700
O2—C7	1.227 (8)	C5—H5B	0.9700
C1—C2	1.364 (8)	C6—H6A	0.9700
C2—C3	1.456 (8)	C6—H6B	0.9700
C2—C7	1.449 (8)	C8—H8A	0.9700
C3—C4	1.508 (9)	C8—H8B	0.9700
C4—C5	1.533 (11)		
			
C1—S1—C8	82.7 (3)	C3—C4—H4B	109.00
C1—S2—C8	83.2 (3)	C5—C4—H4A	109.00
S1—C1—S2	100.5 (3)	C5—C4—H4B	109.00
S1—C1—C2	129.1 (5)	H4A—C4—H4B	108.00
S2—C1—C2	130.4 (5)	C4—C5—H5A	109.00
C1—C2—C3	117.8 (5)	C4—C5—H5B	109.00
C1—C2—C7	119.0 (6)	C6—C5—H5A	109.00
C3—C2—C7	123.2 (6)	C6—C5—H5B	109.00
O1—C3—C2	121.7 (6)	H5A—C5—H5B	108.00
O1—C3—C4	121.1 (5)	C5—C6—H6A	109.00
C2—C3—C4	117.2 (6)	C5—C6—H6B	109.00
C3—C4—C5	112.7 (6)	C7—C6—H6A	109.00
C4—C5—C6	111.5 (6)	C7—C6—H6B	109.00
C5—C6—C7	113.5 (6)	H6A—C6—H6B	108.00
O2—C7—C2	120.7 (6)	S1—C8—H8A	113.00
O2—C7—C6	122.3 (5)	S1—C8—H8B	113.00
C2—C7—C6	116.9 (6)	S2—C8—H8A	113.00
S1—C8—S2	93.6 (3)	S2—C8—H8B	113.00
C3—C4—H4A	109.00	H8A—C8—H8B	110.00
			
C8—S1—C1—S2	1.5 (3)	C1—C2—C3—C4	−178.3 (5)
C8—S1—C1—C2	−179.6 (6)	C1—C2—C7—C6	−178.9 (6)
C1—S1—C8—S2	−1.4 (3)	C3—C2—C7—O2	179.8 (6)
C8—S2—C1—S1	−1.5 (3)	C1—C2—C7—O2	−2.2 (9)
C8—S2—C1—C2	179.6 (6)	C1—C2—C3—O1	2.2 (8)
C1—S2—C8—S1	1.4 (3)	C3—C2—C7—C6	3.1 (9)
S2—C1—C2—C3	−1.6 (9)	C2—C3—C4—C5	24.6 (8)
S1—C1—C2—C3	179.8 (4)	O1—C3—C4—C5	−155.8 (6)
S1—C1—C2—C7	1.6 (9)	C3—C4—C5—C6	−52.6 (8)
S2—C1—C2—C7	−179.7 (5)	C4—C5—C6—C7	56.3 (8)
C7—C2—C3—O1	−179.8 (6)	C5—C6—C7—C2	−31.5 (9)
C7—C2—C3—C4	−0.2 (8)	C5—C6—C7—O2	151.8 (6)

### Hydrogen-bond geometry (Å, º)

**Table:**

*D*—H···*A*	*D*—H	H···*A*	*D*···*A*	*D*—H···*A*
C5—H5*A*···S2^i^	0.97	2.87	3.844 (8)	178

Symmetry code: (i) −*x*, −*y*+2, *z*−1/2.
